# Investigating the protective effects of fluvoxamine against sepsis-related acute lung injury through antiapoptotic, anti-inflammatory, and anti-oxidant features in rats

**DOI:** 10.22038/ijbms.2024.80608.17444

**Published:** 2025

**Authors:** Halil Asci, Suleyman Emre Akin, Hasan Ekrem Camas, Ahmet Bindal, Okan Kurtbolat, Serife Tasan, Abdurrahman Gulal, Rumeysa Taner, Turgut Kurt, Ozlem Ozmen

**Affiliations:** 1 Department of Pharmacology, Faculty of Medicine, Suleyman Demirel University, Isparta, Turkey; 2 Department of Bioengineering, Institute of Science and Technology, Suleyman Demirel University Isparta, Turkey; 3 Department of Thoracic Surgery, Faculty of Medicine, Suleyman Demirel University, Isparta, Turkey; 4 Department of Anesthesiology and Reanimation, Suleyman Demirel University, Isparta, Turkey; 5 Department of Pathology, Faculty of Veterinary Medicine, Burdur Mehmet Akif Ersoy University, Burdur, Turkey; 6 Veterinarian, Isparta, Turkey; 7 Veterinary Technician, Faculty of Medicine, Suleyman Demirel University Isparta, Turkey

**Keywords:** ER stress, Inflammation Lipopolysaccharide, Rat, Respiratory system, SSRI

## Abstract

**Objective(s):**

Acute lung injury (ALI) is characterized by severe hypoxia and alveolar damage, often caused by oxidative stress, endoplasmic reticulum stress (ERS), and apoptosis. Fluvoxamine (FLV), an antidepressant, has tissue-protective properties through various intracellular mechanisms. This study investigates the anti-inflammatory effects of FLV used as an antidepressant in a lipopolysaccharide (LPS)-induced ALI model.

**Materials and Methods:**

Thirty-two female Wistar Albino rats aged 14–16 weeks and weighing 300–350 g, with 8 animals in each group, were divided into four groups: control, LPS, LPS+FLV, and FLV. After LPS administration, rats were euthanized, and histopathological analysis, immunohistochemistry for tumor necrosis factor-α (TNF-α) and caspase-3 (Cas-3), ELISA for oxidative stress markers, and PCR for CHOP, Cas-12, and Cas-9 gene expressions were conducted.

**Results:**

In the LPS group, lung tissue damage, increased inflammatory cell infiltration, increased Cas-3 and TNF-α expressions, increased oxidative stress markers, and increased CHOP, Cas-9, and Cas-12 mRNA expressions were observed compared to the control group. FLV treatment in the LPS+FLV group significantly reversed these effects in the LPS group.

**Conclusion:**

FLV exhibits protective effects against ALI by mitigating inflammation, ERS, and apoptosis via the CHOP/Cas-9/Cas-12 pathway. Further studies are needed to explore additional pathways and potential clinical applications of FLV.

## Introduction

The systemic inflammatory response is a crucial aspect of the body’s immune system, designed to combat external pathogens like bacteria, viruses, and fungi while preventing infections. However, an excessive inflammatory response can lead to tissue damage. This response affects various organs and tissues, including the respiratory system, which can result in sepsis-related acute lung injury (ALI)(1). 

ALI manifests as severe hypoxia and extensive damage to the alveoli, often progressing to acute respiratory distress syndrome (ARDS) and respiratory failure, often with fatal outcomes. The onset of ALI is typically marked by the infiltration of inflammatory cells, particularly neutrophils and macrophages, into the lungs (2). This infiltration triggers the hyperactivation of these inflammatory cells, leading to the excessive release of pro inflammatory cytokines such as tumor necrosis factor-alpha (TNF-α) (3). Additionally, the heightened inflammatory response results in the accumulation of reactive oxygen species (ROS), which are byproducts of oxidative energy metabolism. ROS accumulation further exacerbates the inflammatory response and tissue damage by inducing cell apoptosis through various signaling pathways (4). 

**Figure 1:**
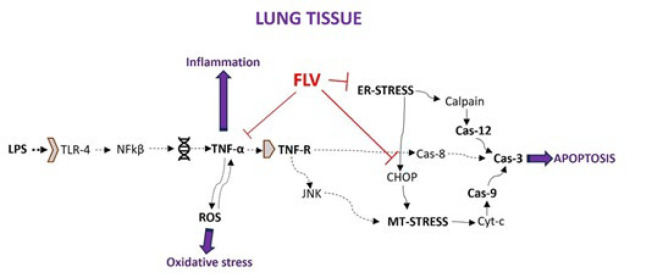
Possible effects of fluvoxamine (FLV) on the lipopolysaccharide (LPS)-induced acute lung injury (ALI) model in rats

In conditions that provoke an inflammatory response, the endoplasmic reticulum (ER), with its essential role in cell viability, may experience stress known as endoplasmic reticulum stress (ERS). ERS can lead to the accumulation of faulty protein structures and trigger the production of caspase-12 (Cas-12), crucial molecules in ER-induced apoptosis (5). Calpain also plays a significant role in Cas-12 activation. ERS-induced Cas-12 activation initiates apoptosis by activating caspase-3 (Cas-3), the final step in the apoptotic pathway mediated by protein kinase R-like ER kinase (PERK)-mediated C/EBP-homologous protein (CHOP). Moreover, ERS can induce apoptosis through mitochondria by upregulating CHOP expression (6, 7). In cases of prolonged ER stress, CHOP is notably upregulated. The activation of mitochondrial stress by CHOP expression decreases antiapoptotic B-cell lymphoma 2 (Bcl-2) levels while increasing apoptotic Bcl-2-associated X-protein (BAX) and Bcl-2 homologous antagonist/killer (BAK) levels. This results in the release of cytochrome C from mitochondria, triggering caspase-9 (Cas-9) activation and ultimately leading to apoptosis associated with Cas-3 activation. Overall, effectively reducing the inflammatory response can halt disease progression (Figure 1)(7, 8). 

Fluvoxamine (FLV) is a selective serotonin reuptake inhibitor commonly prescribed for depression and anxiety disorders. Aside from its serotonin-related effects, FLV has a notable affinity for sigma-1 receptors (S1R) found in cells’ ER (9). Studies have indicated that FLV can mitigate ERS by reducing CHOP expression (10). Moreover, the protective effects of FLV within these mechanisms have been documented (11, 12).

In this study, our objective was to explore the potential beneficial effects of FLV, which influences the intracellular signaling mechanisms discussed above, on the pathophysiology of ALI.

## Materials and Methods

### Ethical approval

The procedures carried out on rats were reviewed and approved by the Animal Experiments Local Ethics Committee of Suleyman Demirel University University (Ethics No: 26.01.2023-121/01). The experiment adhered to the ARRIVE 2.0 guidelines. Additionally, support for the study was provided by the Suleyman Demirel University University Scientific Research Project Unit (SDU-BAP) under project number TSG-2023-9010.

### Chemicals

LPS was obtained from Sigma Aldrich (L2630-100 mg), USA. FLV, Faverin 100 mg, was obtained from Abbott Ltd. Turkey. For sedation and anesthesia induction, Xylazine Bio 2% (Bioveta, Czech Republic) and Keta-Control (Doga Ilaç, Turkey) were utilized.

For immunohistochemical analysis, the Anti-Cas-3 antibody [EPR18297] (ab184787) and the recombinant anti-TNF-α antibody [RM1005] (ab307164) were used as primary antibodies. The mouse and Rabbit Specific HRP/AEC (ABC) Detection IHC Kit (ab93705) was used as a secondary antibody, and aminoethyl carbazole (AEC) was used as chromogen. Primary and secondary antibodies were provided by Abcam (Cambridge, UK). 

For reverse transcription-polymerase chain reaction (RT-qPCR) analysis, RNA isolation from homogenized tissues was performed using GeneAll RiboEx(TM) RNA Isolation Kit (GeneAll Biotechnology, Seoul, Korea). For cDNA synthesis, 1 µg RNA was isolated using A.B.T.™ cDNA Synthesis Kit (Atlas Biotechnology, Turkey). Gene expression levels were evaluated with a 2X SYBR green master mix kit (Nepenthe, Turkey).

### Animals

Thirty-two adult female Wistar albino rats weighing 300-350 g and 14-16 weeks old were housed in standard Euro-type 4 cages, with each group separated from the others. The animals were maintained under controlled environmental conditions, specifically at 55% humidity and 23 ^°^C, following a 12-hour light and 12-hour dark cycle. They were provided *ad libitum* access to standard commercial feed and water. The rats were randomly allocated to groups, and four experimental groups were formed as follows:

*Control group* (n=8): Intraperitoneal (IP) administration of 0.5–1 ml of normal saline (NS) in the left inguinal region of the rats, followed by IP administration of 0.5 ml of NS in the right inguinal region 30 min later.

*LPS group* (n=8): IP administration of 0.5-1 ml of NS in the left inguinal region of the rats, followed by IP administration of 0.5 ml of LPS at a dose of 5 mg/kg in the right inguinal region 30 min later. LPS was dissolved in NS (13).

*LPS+FLV group* (n=8): IP administration of 50 mg/kg FLV in 0.5–1 ml volume of NS in the left inguinal region of the rats, followed by IP administration of 5 mg/kg LPS in 0.5 ml volume in the right inguinal region 30 min later (14).

*FLV group* (n=8): IP administration of FLV at a dose of 50 mg/kg in 0.5–1 ml volume of NS in the left inguinal region of the rats, followed by IP administration of 0.5 ml NS in the right inguinal region 30 min later.

Six hours after the LPS administration, rats were anesthetized with 90 mg/kg ketamine and 10 mg/kg xylazine and then sacrificed. Following the abdominal incision, euthanasia was performed by drawing blood from the inferior vena cava. Lung tissues were removed and portioned. The right lung tissues were placed into a 10% formaldehyde solution for histopathological and immunohistochemical evaluations. The left lung tissues were divided into two equal parts, with one part stored at -80 ^°^C for genetic analysis and the other part stored at -20 ^°^C for biochemical analysis.

### Biochemical analysis

To initiate the experiment, lung tissues were diluted in phosphate-buffered saline (PBS)(10 mM sodium phosphate) with a five-fold weight/volume ratio, adjusting the pH to 7.4. Subsequently, the tissues underwent homogenization using a tissue homogenizer (IKA Ultra Turrax T25, Janke & Kunkel, Staufen, Germany). Following homogenization, samples were centrifuged at 2000 rpm for 20 min at +4 ^°^C using a Nuve NF 1200R centrifuge (Ankara, Turkey). The supernatant obtained after centrifugation was utilized to measure the concentrations of tissue total anti-oxidant status (TAS) and total oxidant status (TOS). An automated biochemistry analyzer (Beckman Coulter AU 5800, Brea, CA, USA) and colorimetric methods developed by Erel were employed for these assays (15, 16). TOS results were expressed as μmol H2O2 Equiv/g, while TAS results were reported as mmol Trolox Equiv/g. The oxidative stress index (OSI) was calculated by dividing TOS levels by TAS levels, denoted as TOS/TAS/10 (17).

### Histopathological analysis

During the necropsy, lung tissue samples were collected and fixed in a 10% buffered formalin solution. After a two-day fixation period, all tissues were routinely processed by an automated tissue processing device and then embedded in paraffin wax. From the paraffin blocks, sections of five-micron thickness were precisely cut using an automated rotary microtome. These sections were then subjected to hematoxylin-eosin (HE) staining and evaluated under a light microscope for detailed histological analysis (18).

The severity of vascular and alveolar changes, edema, and bronchiole pathology were graded on a scale from 0 (normal) to 3 (severe). The examined features included pulmonary congestion, inflammation, thickening of the alveolar septal tissue, and detachment of the bronchiole epithelium, following modifications by Passmore *et al*. (19). Histopathological scoring criteria are provided in Table 1. 

### Immunohistochemical analysis

Moreover, following the manufacturer’s guidelines, two sets of sections were extracted from each paraffin block and mounted on poly-L-lysine-coated slides for immunohistochemical staining targeting TNF-α and Cas-3 expression. Specifically, these sections were stained using -Cas-3 antibody and TNF-α antibody, with each primary antibody diluted to 1/100. After overnight exposure to the primary antibodies, immunohistochemistry was performed using a streptavidin-alkaline phosphatase conjugate and a biotinylated secondary antibody. The Mouse and Rabbit Specific HRP/AEC (ABC) Detection IHC Kit (ab93705) served as the secondary antibody, and aminoethyl carbazole (AEC) was employed as the chromogen. Both primary and secondary antibodies were supplied by Abcam (Cambridge, UK). Negative controls utilized antigen dilution solution in place of primary antibodies. Evaluation of the samples was conducted by a trained pathologist from another university in a blinded manner.

The percentage of immune-positive cells for each marker in 10 distinct fields on each slide for all groups was calculated at an objective magnification of X40. Image analysis was performed using the ImageJ program (National Institutes of Health, Bethesda, MD, version 1.48). Images were processed by separating them into color channels, cropping, and eliminating any artifacts. Cells within the regions of interest were initially selected using a selection tool, and then the software’s counting tool was employed. Strong red staining was used to identify positively stained cells. Microphotographs were captured using the Database Manual Cell Sens Life Science Imaging Software System (Olympus Co., Tokyo, Japan)(20, 21)

### Reverse transcription-polymerase chain reaction (RT-qPCR)

RNA isolation from homogenized tissues was performed using the GeneAll RiboEx (TM) RNA Isolation Kit (GeneAll Biotechnology, Seoul, Korea) following the manufacturer’s protocol. The quantity and purity of the obtained RNAs were assessed using the BioSpec-nano nanodrop device (Shimadzu Ltd., Kyoto, Japan). For cDNA synthesis, 1 µg of RNA was utilized with the A.B.T. ™ cDNA Synthesis Kit (Atlas Biotechnology, Turkey) in a thermal cycler as per the provided protocol. Primer sequences were designed based on specific mRNA sequences, and potential primer sequences were evaluated using the NCBI website. The primer sequences used are detailed in Table 2. Gene expression levels were evaluated using a Biorad CFX96 real-time PCR instrument (California, USA) with 2X SYBR green master mix (Nepenthe, Turkey). The GAPDH gene served as the housekeeping gene in the study. The reaction mixture, prepared according to the kit manufacturer’s protocol, reached a final volume of 20 µl. This mixture was loaded into a real-time qPCR device following the kit’s instructions, and each sample was analyzed in triplicate. PCR conditions consisted of an initial denaturation at 94 °C for 10 min, followed by 40 cycles of denaturation at 95 °C for 15 sec, and annealing/extension at 55 ^°^C for 30 sec. Relative mRNA levels were determined by applying the 2-^ΔΔCt^ formula to the normalized results.

### Statistical analysis

For statistical analysis, we used the GraphPad Prism 10 (Version 10.1.0) (GraphPad Software, USA) program. Initially, the data were analyzed for normality of distribution using the Shapiro-Wilk test. Since the data showed a normal distribution (*P*>0.05), comparisons between the groups were made with one-way analysis of variance (ANOVA). The Tukey test was used to identify differences between groups, with values of *P*<0.05 considered statistically significant. For nonparametric data, the Mann-Whitney U test and Dunnett’s C test were used to detect differences between groups.

## Results

### Biochemical results

**Figure 2:**
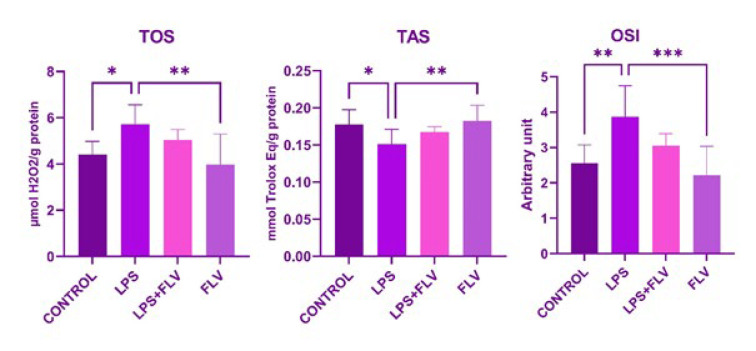
Statistical analysis of oxidative stress parameters of this study

In the LPS group, there was a significant increase in TOS values and a decrease in TAS values compared to the control group (*P*=0.023 and *P*=0.031, respectively). The OSI values in this group showed a significant increase compared to the control group (*P*=0.003). In the LPS+FLV group, although TOS and OSI values increased and TAS values decreased compared to the control group, these changes were not statistically significant (*P*>0.05). Conversely, in the FLV group, there was a significant decrease in TOS and OSI values compared to the LPS group (*P*=0.001 and *P*<0.001, respectively). Additionally, there was a notable increase in TAS values in the FLV group compared to the LPS group (*P*<0.008), as illustrated in Figure 2.

### Histopathological results

**Figure 3:**
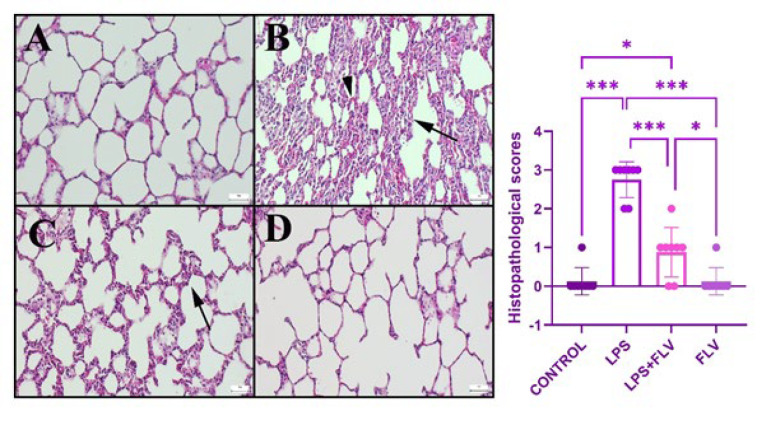
Histopathological appearance of lungs among the groups of rats

The lung histopathology of both the Control and FLV groups revealed normal tissue appearance. In the LPS group, marked hyperemia, increased septal tissue thickness, hemorrhage, and inflammatory cell infiltrations were observed compared to the control group. The pathological findings in the LPS+FLV group were mitigated through FLV treatment, as depicted in Figure 3.

### Immunohistochemical findings

**Figure 4:**
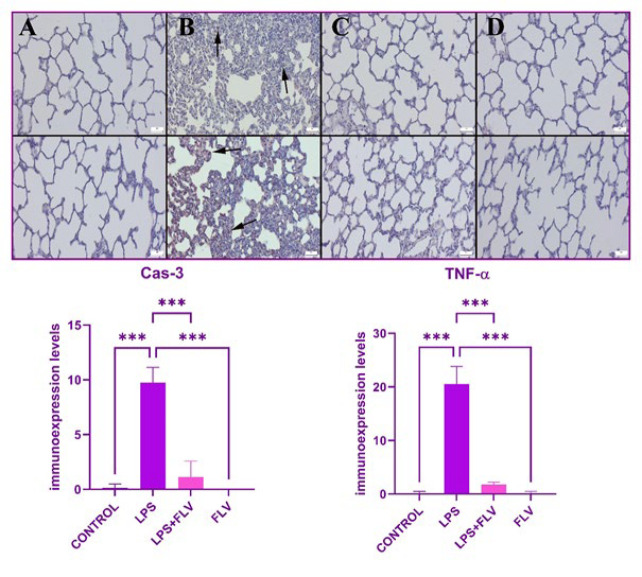
Immuno expressions of Cas-3 (upper row) and TNF-α (below row) in lung tissues of rats

Slides from the control group’s immunohistochemical staining revealed either no or very little Cas-3 and TNF-α expression. Cas-3 and TNF-α expressions significantly increased in the LPS group (*P*=0.001 for both). After FLV therapy, Cas-3 and TNF-α expression also significantly decreased (*P*=0.001 for both). The FLV group’s expressions matched those of the control group with markers in terms of similarity. Inflammatory cells, alveolar macrophages, and alveolar epithelial cells often exhibited expressions. The results of a statistical analysis of immunohistochemistry expressions are shown in Figure 4.

### Genetic results

**Figure 5:**
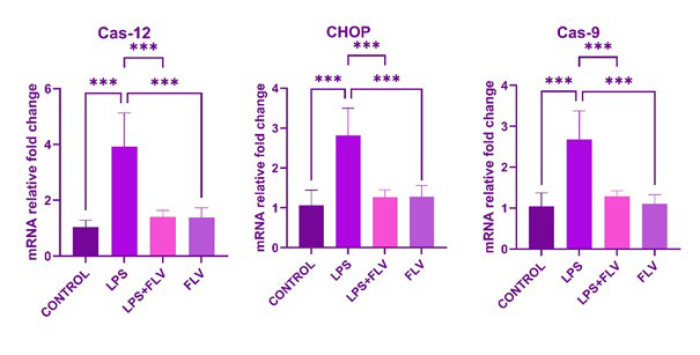
Expression levels of Cas-9, Cas-12, and CHOP in lung tissues of rats

**Figure 6:**
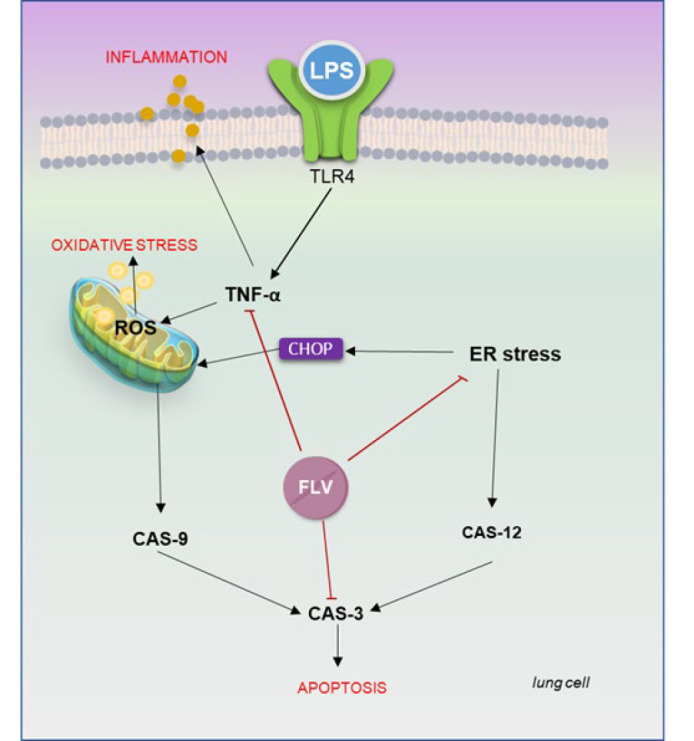
Probable mechanism of Fluvoxamine’s healing effect on lipopolysaccharide (LPS)-induced lung injury of rats

In the LPS group, the expression levels of Cas-9, Cas-12, and CHOP exhibited a significant increase compared to the control group (*P*=0.001 for all). However, in the LPS+FLV group, treatment with FLV led to a significant reversal of these parameters compared to the LPS group (*P*=0.001 for all). Notably, in the FLV group alone, the levels of Cas-9, Cas-12, and CHOP showed a significant decrease compared to the LPS group (*P*=0.001 for all)(Figure 5). The probable mechanism of FLV’s healing effect on LPS-induced lung injury is shown in Figure 6.

## Discussion

In this study, we aimed to investigate the potential protective effects of FLV against sepsis-associated ALI through modulation of apoptotic and inflammatory pathways and ER stress. Sepsis is a life-threatening condition characterized by a dysregulated host response to infection, often leading to multiple organ dysfunction, with the lungs being particularly susceptible. ALI, a common complication of sepsis, involves inflammation, oxidative stress, and apoptosis, contributing to tissue damage and respiratory dysfunction. FLV, an antidepressant known for its anti-inflammatory and anti-oxidant properties, has recently garnered attention for its potential therapeutic effects beyond psychiatric disorders. However, its role in mitigating sepsis-induced lung injury remains underexplored. By investigating the apoptotic pathways modulated by FLV in the context of sepsis-related ALI, we aim to contribute to a deeper understanding of its potential as a therapeutic agent in critical care settings.

In diseases like sepsis, which can affect multiple organs, brain tissue damage may occur due to increased blood-brain barrier permeability caused by the heightened systemic inflammatory response aimed at combating the bacterial agent, while highly vascularized tissues such as the heart and lungs can also suffer significant damage (22). When both the brain and peripheral organs are affected, patients often exhibit severe clinical symptoms that complicate treatment, and inadequate protocols may result in peripheral organ damage along with psychiatric conditions like depression, especially in intensive care patients (23-26). This damage is linked to oxidative stress and inflammation caused not only by the bacterial agent but also by pro-oxidant and pro inflammatory substances circulating in the bloodstream, which target specific receptors in these tissues (22, 27). More serious mechanisms, such as apoptosis, leading to cell death, may also be triggered, contributing to conditions like depression (28-32). Antidepressants like FLV, which possess anti-inflammatory and anti-oxidant properties in addition to their primary therapeutic effects, offer additional benefits in treatment (33-35). This study explores the protective effects of FLV on lung tissue, focusing on its potential to mitigate organ damage beyond the central nervous system.

It is known that oxidative stress is also provoked in lung tissue in LPS-induced damage, which serves as a model for systemic inflammatory response (36-40). According to the biochemical analyses in this study, the increased TOS and decreased TAS values in the LPS group indicate the development of oxidative stress in lung tissue. The observation that these values were partially reversed with FLV treatment, albeit not significantly, may be attributed to insufficient drug administration. This warrants further investigation in other studies by exploring higher doses or repeated drug administration. Additionally, the fact that the FLV alone group did not decrease the levels of oxidant substances or significantly increase the levels of anti-oxidant enzymes compared to the control group suggests the involvement of other mechanisms.

Oxidative stress is known to trigger inflammation through the activation of certain intracellular pathways. Considering the histopathological and immunohistochemical findings in this study, it can be inferred that such activation occurred. The marked hyperemia, increased septal tissue thickness, inflammatory cell infiltrations, and heightened TNF-α expressions observed in the lung tissues of the LPS group indicate the development of inflammation. The significant reversal of these parameters with FLV treatment demonstrates the potential protective effect of the drug on lung tissue. Moreover, it appears that the anti-inflammatory activity takes precedence over the anti-oxidant activity in this protection. The ability of FLV to suppress the increased TNF-α levels in lung tissues induced by acute inflammation, even with a single dose, suggests its potential utility in similar acute events. There exists a close association between inflammation and apoptosis, a more serious condition. TNF-α released from inflammatory cells at the site of damage is known to activate its own receptor-mediated intracellular pathways, leading to apoptosis by upregulating the expression of Cas-3, the final step in these pathways (41-50). The increased TNF-α expressions detected in immunohistochemical analysis, along with the parallel increase in Cas-3 expression in alveolar macrophages, inflammatory cells, and alveolar epithelium, highlight this scenario. Changes in Cas-3 expression may be mediated by ERS and mitochondria or directly by TNF-α.

The intracellular signaling mechanism activated by TNF-α stimulation can trigger caspase-8-mediated Cas-3 activation, as well as stimulate mitochondria-mediated Cas-9 and Cas-3 expressions by increasing JNK expressions. Furthermore, while Cas-12-mediated Cas-3 expression may be directly increased as a result of ERS, mitochondria may be influenced by CHOP signaling, and Cas-9-mediated Cas-3 expression may be triggered, ultimately resulting in apoptosis (51-60). In this study, the increase in Cas-12 expressions detected in the lung tissues of the LPS group indicates the involvement of ERS. In contrast, the increase in CHOP and Cas-9 expressions indicates mitochondrial stress. The fact that FLV leads to a decrease in both TNF-α and these expressions suggests that the drug can mitigate apoptosis secondary to inflammation or directly.

Furthermore, the scarcity of literature demonstrating the antiapoptotic properties of FLV, especially its effects on lung tissue, underscores the innovative aspect of this study. Limitations of this study include the use of a single drug dose, lack of testing for different doses, and absence of protein-level support for genetic analyses with western blot analyses.

**Table 1:** Scoring criterion for pulmonary histopathological lesions

Score	Vascular features	Extravascular alveolar involvement	Bronchiole features
0	Normal	None	None
1	Slight hyperemia	Mild edema and inflammatory reaction	Mild infiltration of inflammatory cells
2	Moderate hyperemia	Moderate inflammatory reaction and areas of moderate alveolar thickening (25-50% visualized lung)	Moderate infiltration of inflammatory cells; detachment of lining in some bronchioles
3	Severe hyperemia	Moderate-severe inflammatory exudate and severe alveolar thickening (> 50% visualized lung)	Complete loss of bronchiole structure; detachment of lining; cellular debris and inflammatory cell exudate

**Table 2:** Primary sequences, product size, and accession numbers of genes that used in this study

Genes	Primary sequence	Product size	Accession number
GAPDH (HouseKeeping)	F: AGTGCCAGCCTCGTCTCATA	248 bp	NM_017008.4
	R: GATGGTGATGGGTTTCCCGT		
Caspase 9	F: AGCCAGATGCTGTCCCATAC	148 bp	XM_039110693.1
	R: CAGGAACCGCTCTCTTCTTGTC		
Caspase 12	F: CTGCATCAGAATCCAGGGGA	212 bp	NM_130422.1
	R: TCGGCCTTCCTTCCTTCTCCATCA		
CHOP	F: TGGAAGCCTGGTATGAGGATCTG	175 bp	XM_006241445.4
	R: GAGGTGCTTGTGACCTCTCTGCTG		

F: Forward, R: Reverse, GAPDH: glyceraldehyde-3-phosphate dehydrogenase, CHOP: C/EBP homologous protein

Values are presented as means±standard deviation. A one-way ANOVA test was used. * *P≤*0.05, ** *P≤*0.01, *** *P≤*0.001

LPS: Lipopolysaccharide, FLV: Fluvoxamine, TAS: Total anti-oxidant status, TOS: Total oxidant status, OSI: Oxidative stress index

(A) Normal lung histology in the control group. (B) Hyperemia (arrowhead) and increased septal tissue thickness (arrow) in lungs in the LPS group. (C) Decreased septal tissue thickness (arrow) and pathological findings in the LPS+FLV group. (D) Normal tissue architecture of the FLV group, HE, scale bars=50 µm. Values are presented as means±standard deviation. A one-way ANOVA test was used. * *P≤*0.05, ** *P≤*0.01, *** *P≤*0.001

LPS: Lipopolysaccharide, FLV: Fluvoxamine

(A) Negative expression in the control group. (B) Marked increase in (arrows) in LPS group. (C) Decreased expression in the LPS+FLV group. (D) No immuno expression in the FLV group. Streptavidin biotin peroxidase method, scale bars=50 µm. Values are presented as means±standard deviation. A one-way ANOVA test was used. * *P≤*0.05, ** *P≤*0.01, *** *P≤*0.001

LPS: Lipopolysaccharide, FLV: Fluvoxamine, Cas-3: Caspase-3, TNF-α: Tumor necrosis factor-alpha

Values are presented as means±standard deviation. A one-way ANOVA test was used. * *P≤*0.05, ** *P≤*0.01, *** *P≤*0.001

LPS:Lipopolysaccharide, LPS+FLV: Lipopolysaccharide+Fluvoxamine, FLV: Fluvoxamine, Cas-9: Caspase-9, Cas-12: Caspase-12, CHOP: C/EBP homologous protein

## Conclusion

This study has demonstrated that FLV, an effective antidepressant, suppresses the inflammatory and apoptotic processes induced by systemic inflammation in lung tissue. Future studies should focus on conducting more detailed analyses of this activity, including exploring different intracellular pathways. It would be advisable to complement such molecular research with clinical studies for a comprehensive understanding of FLV’s therapeutic effects in the context of lung inflammation.

## Declaration

We have not used any AI tools or technologies to prepare this manuscript.
